# Evaluation of a Spanish Health Topics Course for Undergraduate Pre-health Latino Students

**DOI:** 10.7759/cureus.5825

**Published:** 2019-10-02

**Authors:** Pilar Ortega, Yoon Soo Park, Alicia J Rodriguez, Jorge A Girotti

**Affiliations:** 1 Medical Education, University of Illinois, Chicago College of Medicine, Chicago, USA; 2 Miscellaneous, Arturo Velásquez Institute of Richard J. Daley College, Chicago, USA

**Keywords:** hispanic/latino health, premedical education, pipeline programs, medical spanish, language concordance, premedical education, limited english proficiency, limited english proficiency, pipeline programs, underrepresented minorities, language concordance

## Abstract

Introduction: Language concordance between doctors and patients and increased matriculation of underrepresented minorities in medical school are commonly cited goals of medical centers and medical schools in addressing health disparities for individuals with limited English proficiency. Pre-medical education may represent a high-yield untapped opportunity to address both through a Spanish Health Topics, or *Temas de Salud*, course for Latino pre-health students.

Methods: The authors implemented a longitudinal course for pre-health, Latino, undergraduate students to prepare them for success in bilingual health careers. The course included educational components of health knowledge, Spanish terminology, Hispanic/Latino sociocultural context, and exposure to medical learning formats presented during monthly sessions spread out over two years. A post-course survey with comfort and knowledge assessments was administered after each one-year cycle of the program.

Results: One hundred and sixteen students (57%) out of 203 course-participants responded to the Spanish Health Topics course survey. The student comfort level and self-perceived knowledge about specific health issues increased for both students of native-level Spanish and less advanced fluency, though a larger improvement was noted in several health topics for native speakers. Eighty-five percent of students reported perceiving the class to be useful for their future careers, and 92% of respondents indicated having applied learned concepts in social and/or academic settings outside of class.

Discussion: Most students reported benefits of the course. Future studies should focus on a more detailed evaluation of enrolled students’ knowledge, attitudes, confidence, and long-term retention compared to students in a standard premedical path. *Temas de Salud* may enhance the bilingual, bicultural skillset of Latino underrepresented minorities in medicine, and can be replicated at other institutions.

## Introduction

Medical centers struggle with the challenge of increasing underrepresented minority representation in their health workforce to better reflect their patient populations. Underrepresented minority populations have reduced access to primary care and specialty health providers. Providers who are also underrepresented minorities are more likely to serve medically underserved communities as compared to their white counterparts [1].

Linguistically and culturally concordant care is considered to be optimal for patient satisfaction and quality of care [2,3]. Hispanic/Latinos (abbreviated henceforth as “Latinos”) are significantly underrepresented in medical careers [4]. Recent national data shows that 64% of the U.S. limited English-proficient population is Spanish-speaking, whereas all other languages each comprise between 1-6% [5]. However, there are considerably fewer Latino physicians compared to the growing number of Latino patients. A recent study showed that in the past 30 years, the number of Latino U.S. physicians per 100,000 people has declined by 22% [4].

One important factor in the decline of Latino physicians is the lack of Latino students that are pursuing higher academic levels within the sciences, including careers in Medicine. Several studies have looked at multifactorial issues associated with underrepresented minorities’ declining interest over time in premedical education or research-focused careers, and worse performance on examinations compared to nonminority peers [6-9]. Some programs have had success in implementing pipeline programs that emphasize health disparities education and research development, as well as multicultural education that provides a social context of health that is distinct from biomedical science-focused education [10-12].

To date, no studies have formally evaluated student learning outcomes pertaining to language skills in premedical undergraduates who aim to serve a focused underserved population, such as the growing Spanish-speaking U.S. population. Regardless of their cultural background or knowledge-base, providers who treat the Latino population may have never been exposed to communication skills education to address the healthcare needs of this patient population in their preferred language. Medical Spanish programs exist at some medical institutions but mostly target practicing health professionals, medical students, or residents [13].

Some, but not all Latino pre-medical students may be considered Spanish heritage speakers-individuals who have learned Spanish as a first or second language at home, though may have significant variability in both their general Spanish language proficiency and even more so in their healthcare linguistic competency [14]. As a result, pre-medical students who are also Spanish heritage speakers may be asked to serve as ad hoc interpreters without ever having had any formal health-related or Spanish-language training. This practice has been reported to occur in a recent study involving medical students but has never been studied in pre-health undergraduates who frequently serve as volunteers, scribes, or other roles in medical centers [15]. An untrained interpretation has been shown to result in reduced quality of medical interpretation and physician-patient communication [3]. Although some researchers have evaluated the accuracy of self-reported language proficiency in medical students, and recent work has prompted a national call-to-action to increase attention to language concordance and language assessment in medical education, no data is available for healthcare Spanish training programs earlier training, such as for undergraduate pre-health students [16,17]. 

A few programs promoting Spanish language and culture curricula and service-learning components within undergraduate pre-health programs in Hispanic-serving institutions have been described, but data has not been published regarding program outcomes such as student performance, attitudes, or comfort level [14,18]. Such pre-health programs serve the dual objective of increasing student medical school preparedness and of enhancing student cultural and language skills. By engaging pre-health undergraduates in a course that provides some clinical education relevant to their desired career goal, pipeline programs hope to achieve increased retention of these underrepresented students as medical professionals. The integration of language and cultural training aims to increase the eventual language concordance and communication skills of these future physicians with Spanish-speaking patients. Introducing undergraduate pre-health students to Spanish-language health care vocabulary and concepts is a potentially powerful first step in supporting the development of linguistically and culturally competent future health care providers. This novel approach to early language exposure and training may not only help to adequately prepare students for the linguistic challenges they will encounter as medical students, residents, and physicians, but it may also allow more time later in training for more advanced skill development. 

The purpose of our innovative pilot program is to describe and explore the knowledge and attitudinal changes among pre-health undergraduates exposed to a clinically focused health topics curriculum in Spanish, simultaneously presenting students with health information and Spanish-language skills development.

## Materials and methods

Medicina Scholars is an existing program of the Hispanic Center of Excellence (HCOE) at the University of Illinois Chicago - College of Medicine with the goal to increase the overall retention of these students in medical careers and their ability to care for Latino patients. Thirty undergraduate Latino pre-health students per academic year are accepted into a three-year longitudinal program consisting of academic advising and a Saturday lecture series curriculum. After several years of implementation, it was recognized that this enrichment program for undergraduate students was not adequately addressing one of the key aims of the HCOE, namely, preparing the students linguistically for serving the Latino community. In response to this gap, the *Temas de Salud *course was designed to prepare students for success in bilingual health careers through an educational program that integrated Spanish linguistic skills. 

**Figure 1:**
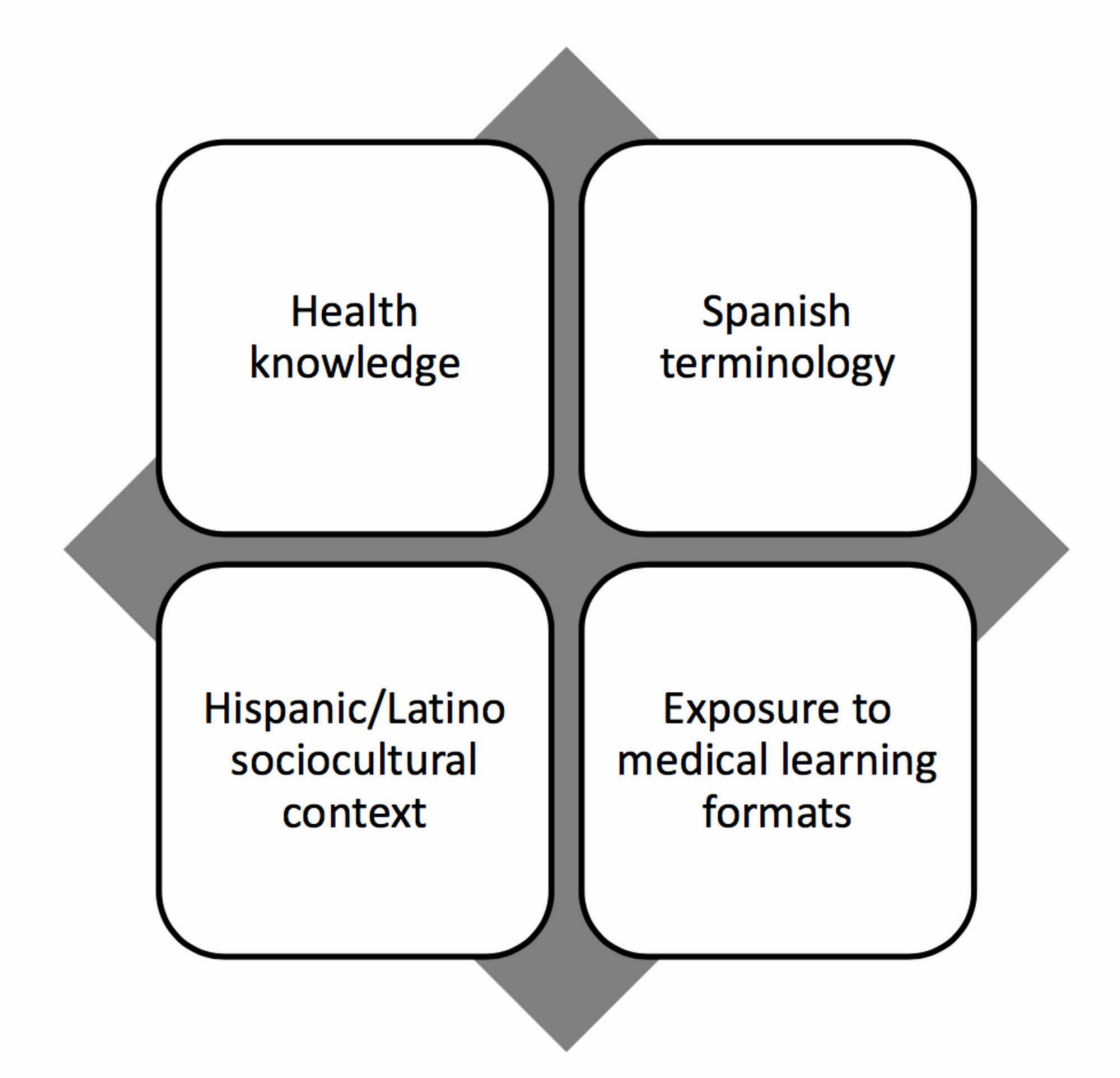
Four Components of the Temas de Salud Course

To address the linguistic component, starting in 2015, the authors implemented a faculty-taught longitudinal *Temas de Salud* (“Spanish Health Topics”) course to introduce pre-health track undergraduates to health-related language in Spanish. All participating students were enrolled in the Medicina Scholars program. The course was designed to increase the Spanish health knowledge base for the Medicina Scholars students and to familiarize the students with key components of future medical training. The course uses Spanish-language immersion to target basic health knowledge acquisition about specific topics, familiarize students with Spanish medical terminology, introduce the relevance of the Latino sociocultural context to health, and expose learners to medical learning formats. The four key components of the course are depicted in Figure 1.

By addressing health concepts in Spanish at a level appropriate to pre-health students, the course aimed that by the end of the course, each student would: Understand basic clinically-relevant healthcare, including anatomy, physiology, common diseases, and treatments for focused, common health topics; Understand and be able to utilize Spanish-language health-related terminology; Understand the social and cultural context of the given health topic for the Latino population; Gain exposure to multiple learning formats that the students must master to succeed in a medical school, such as lectures, problem-based learning activities, multiple-choice examinations, and patient-education activities. A breakdown of the two-year longitudinal curriculum is presented in Table 1. The “Language & Health” overview topic is taught as the first session at the beginning of each academic year to provide all new and returning students with an introduction or review of linguistic diversity, the relation of language to health care, and the role of students and physicians in delivering linguistically equitable care. All the material is taught in Spanish in an immersion-like approach, but students are encouraged to ask questions to clarify any new terminology or concepts, and combined Spanish/English explanations are provided as needed to integrate terms or ideas that students may have previously learned in English in other educational settings. All the learning activities are framed within the context of the health topics presented. 

**Table 1:** Curriculum Summary for Spanish Health Topics Course

Breakdown of Topics By Year for 2-year Longitudinal Spanish Health Topics (Temas de Salud) Curriculum		Breakdown of Class Activities for Each Topic: Typical 2-hour class session
Year 1	Topic A. Language & Health, Cultural focus: Impact of language discordance	10 min. Large Group Activity Ice-breaker activity and quick review of prior session’s teaching points.
	Topic B. Cardiovascular Disease, Cultural focus: Preventive care	
	Topic C. Diabetes, Cultural focus: Myths in treatment of diabetes	50 min. Lecture-based new material Faculty-led presentation on the given health topic in Spanish with an emphasis on Latino patient care and cultural issues.
	Topic D. Cancer, Cultural focus: Barriers to cancer screening	
	Topic E. Depression & Mental Health, Cultural focus: Stigma of mental illness	30 min. Small Group Activity: Problem Based Learning Students small groups to work through a case; case description, guided questions, and discussion are in Spanish.
Year 2	Topic A. Language & Health, Cultural focus: Impact of language discordance	
	Topic B. Tobacco, Alcohol & Substance Use, Cultural focus: Strategies for community education	20 min. Large Group Activity Students orally present their responses in Spanish to the cases in a large group setting followed by discussion and faculty-led summary of learning points.
	Topic C. Dementia, Cultural focus: Caregiver health	
	Topic D. Sexual & Reproductive Health, Cultural focus: Pregnancy planning	10 min. Student feedback and quiz
	Topic E. Hypertension & Kidney Disease, Cultural focus: End-stage renal disease	

After the first two years of *Temas de Salud* course implementation, the authors are interested in evaluating the perceived change in student comfort level with discussing health topics in Spanish, their perceived knowledge acquisition, and their performance in a post-course knowledge assessment examination. 

Participants

Students were invited to participate in the study if they were undergraduate students enrolled in the Medicina Scholars program of the Hispanic Center of Excellence at the University of Illinois at Chicago College of Medicine over a two-year period from 2015-2017. Data were collected for 116 out of 203 (response rate 57%) students. For the 2015-2016 academic year, data were collected for 56 students (55% response rate) and for 60 students (59% response rate) in the 2016-2017 academic year. Students were invited to participate in the survey regardless of undergraduate year, and there was no Medicina Scholar program student attrition.

Data collection

Participating students were asked to complete an electronically submitted voluntary post-course survey at the conclusion of each one-year cycle of the longitudinal *Temas de Salud* course. The survey asked the students to self-rate their comfort level and knowledge acquired in various categories relevant to the material presented in the course using a Likert scale, as well as to rate their opinion of course effectiveness. The survey instrument, including a proficiency self-rating scale, was based on the instrument used at the institution’s medical Spanish course for medical students with adaptations appropriate to the undergraduate level and to the health topics that were being discussed [13]. 

Students were also asked to answer knowledge questions pertaining to the course material as an objective assessment of the material retained several months after course lectures. Knowledge questions were created by the faculty member teaching the course and feedback was provided by the Medicina Scholars’ graduate assistants and faculty to ensure the questions were appropriate for students. Questions were based on the material that had been presented and/or practiced during lectures and small group activities. A select portion of the Spanish Health Topics course's assessment questions regarding self-reported proficiency and perceived change in knowledge/comfort questions are attached as appendices to facilitate a better understanding of the study and easier replication at other institutions.

To facilitate substantive data analysis, we asked respondents to provide their gender, nationality of Latino descent, year in college, and any prior Spanish exposure or courses they have completed. The institutional review board of the University of Illinois approved this study.

Measures

Descriptive statistics were calculated to examine overall trends in students’ survey responses. We used t-tests to compare means and used chi-squared tests to compare proportions and frequencies. Knowledge assessment scores are presented as the percentage of responses correct. The increase in comfort level and self-reported knowledge was also measured. The results were analyzed as differences in performance for students of varying proficiency levels. Significance was reported for p < .05, and p < .01. Data compilation and statistical analyses were conducted using Stata 14 (StataCorp LLC, College Station, TX).

Self-rated proficiency scales are commonly used in medical settings to guide learning activities targeting pre-course language level for medical students and physicians interested in gaining medical Spanish training [13]. The proficiency scale used in the Temas de Salud course allows respondents to choose between six proficiency categories with brief descriptions corresponding to none, beginner, low-intermediate, mid-intermediate, high-intermediate/advanced, and native proficiencies.

Outcome measures included: self-perceived comfort level improvement with specific health topics, perceived knowledge improvement with regards to specific course components, reported skills application outside of class, perceived utility of course-organizational elements (e.g., lecture, small group activities, large group activities), and student performance on post-course knowledge assessment questions as a measure of long-term knowledge attained. In the analysis of Likert-scale outcomes of comfort level improvement, our results will report the "strongly agree" category separately from the other responses in order to better tease out the potential differences in learning for learners with subtle distinctions in Spanish proficiency.

## Results

Over two years, 116 out of 203 students (57%) participated in the *Temas de Salud *course survey. Participating student undergraduate status consisted of 14% freshmen, 20% sophomore, 30% junior, and 30% senior. All enrolled students self-assessed their proficiency level as intermediate level or above. About 47.4% (55 students) classified their language skills at a native level, described as “just as comfortable conversing in Spanish as in any other language.” From the remaining students who self-assessed in intermediate categories, the majority (48 students, or 41.4% of respondents) identified as advanced-intermediate, whereas only 7.6% (nine students) identified as mid-intermediate, and 3.4% (four students) as low-intermediate. Thirty-six percent of students reported growing up with a home language of Spanish only, 37% primary Spanish-secondary English bilingual household, and 20% primary English-secondary Spanish bilingual household. Self-reported nationality distribution among the students consisted of 85% México, 6% Ecuador, 3% Puerto Rico, and 3% Colombia. Seventy-two percent of the participants were female, and the majority of students indicated their health career goal as physician (83%) with a few students indicating nurse practitioner (4%) and Ph.D. researcher (3%) as their goals. Students indicated limited prior exposure to the material presented in the course, with the majority of pre-course knowledge coming from sources such as family and peers. Only 10% of students indicated they had had any previous experience with problem based-learning prior to the Temas de Salud course. 

Eighty-five percent of participants reported attending the language topic course, 41% attended the diabetes course, 38% attended the depression course, 33% attended the cancer course, 46% the tobacco and alcohol course, 45% the dementia course, 35% sexual health, and 34% hypertension, with an average attendance rate of 42% for each session.

Table 2 shows the score reports from a summative knowledge assessment examination that students completed at the end of the course. The displayed results are separated by content-area covered in the *Temas de Salud* course to identify student performance in different topic areas. Scores represent the percentage of questions that were answered correctly by each student, and the results are shown for students at native versus intermediate proficiency levels; the intermediate proficiency includes the low, mid, and advanced-intermediate subcategories described above.

**Table 2:** Knowledge Assessment Score Distribution by Content Area Note: p-values based on t-test Note: Scores represent percentage correct responses on knowledge assessment examination.

Area Assessed	Intermediate Proficiency			Native Proficiency			Overall			p-value
	n	Mean	SD	n	Mean	SD	n	Mean	SD	
Language & Health overview	52	54	3	50	68	3	102	61	3	.002
Diabetes	27	47	5	23	46	6	50	47	4	.527
Cardiovascular	27	59	4	23	61	4	50	60	3	.391
Depression	27	89	4	23	83	5	50	86	3	.847
Cancer	27	63	6	23	71	6	50	67	4	.169
Tobacco & alcohol	25	87	4	27	88	6	52	87	4	.450
Dementia	25	60	6	27	64	5	52	62	4	.297
Sexual health	25	83	4	27	85	4	52	84	3	.330
Hypertension and kidney	25	56	5	27	56	6	52	56	4	.522
Total	53	67	2	50	71	2	103	69	1	.074

Table 3 depicts the self-reported increase in student comfort level and knowledge about specific health issues discussed in the course as varied by student’s baseline Spanish proficiency level. The table displays the percentage of students who strongly agreed that the course improved their comfort level in discussing the indicated health topics. On average, only a small percentage (3.1%) of students disagreed or strongly disagreed with this statement for any of the health topics. To better tease out the differences in learning for learners with more subtle distinctions in Spanish proficiency, we, therefore, have displayed the "strongly agree" category separately from those who selected "agree." While most students reported agreeing or strongly agreeing, on a Likert scale, that the course improved their comfort and knowledge about each health topic, areas such as diabetes, mental health, and dementia demonstrated a larger improvement for students with native-level proficiency compared to those with lower self-reported proficiency levels. Less advanced students reported increased utility, on a Likert scale, of the presentation handouts for their knowledge improvement (82% non-native speakers versus 51% native speakers, P < .001). 

**Table 3:** Increase in Comfort Level and Self-reported Knowledge by Language Proficiency: Proportion (%) of Strongly Agree Note: *p< .05, **p< .01; p-values based on χ2test

Content	Item	Intermediate Proficiency	Native Proficiency	Total	p-value
Temas de Salud increased my comfort level in the following Health Topics in Spanish	When medical interpreter is needed	62.07	71.70	66.67	.282
	What is diabetes?*	41.38	69.57	53.85	.043
	How to improve risk of diabetes**	37.93	78.26	55.77	.004
	Complications of diabetes**	31.03	69.57	48.08	.006
	What is cardiovascular disease?	40.74	52.38	45.83	.422
	What is cholesterol?	33.33	59.09	44.90	.071
	What is mental health?*	44.44	73.68	56.52	.049
	Causes of mental disorders	48.15	55.56	51.11	.626
	What is depression?	44.44	68.42	54.35	.108
	What is cancer?	54.17	50.00	52.50	.796
	What does cancer stage mean?	54.17	50.00	52.50	.796
	Recommended cancer screening	50.00	56.25	52.50	.698
	Consequences of tobacco use*	53.85	80.00	67.86	.037
	Consequences of alcohol use	59.26	80.00	70.18	.087
	What is dementia?*	47.83	79.31	65.38	.018
	What to do if you suspect dementia	60.87	65.52	63.46	.730
	Cultural norms for sexual health	61.54	80.77	71.15	.126
Temas de Salud increased my knowledge	Medical sexual health terminology	54.17	64.00	59.18	.484
	Prevalence of sexually transmitted infections/teen pregnancy	65.38	57.69	61.54	.569
	What is blood pressure?	58.33	62.07	60.38	.782
	Consequences of hypertension	60.00	62.07	61.11	.876
	What is kidney disease?	54.55	60.00	57.45	.706
	Overall health terminology	63.79	64.81	64.29	.910
	General health knowledge	74.14	72.22	73.21	.819
	Cultural knowledge	72.41	79.63	75.89	.372
	Patient communication skills	68.97	66.67	67.86	.795
	Info about common diseases	67.24	68.52	67.86	.885
	Healthy lifestyle/prevention choices	70.18	72.22	71.17	.812

Eighty-five percent of all respondents indicated strong agreement with the statement that the class adequately introduced them to key health topics in Spanish that will be useful in their intended career. Moreover, a vast majority (92%) of respondents indicated that they had had an opportunity to apply the skills taught in the Spanish Health Topics course in various social and academic settings; 72% reported using the learned skills with their family, 45% indicated applying the skills with friends, 30% used them in clinical settings (such as volunteering or shadowing experiences), 23% used the skills at school, and 16% at work.

## Discussion

Initial data suggests that there are some differences in the outcomes among Spanish heritage speakers based on their starting proficiency level. For example, native speakers have a greater capacity to increase their knowledge base based on oral lectures, whereas less advanced speakers report greater benefit from handouts and written materials that may help them to review or reinforce the material outside of class time. Most of their knowledge assessment scores did not differ significantly based on proficiency level. 

A few topic areas, however, did display some knowledge differences with more fluent speakers performing better on the assessment, such as mental health, dementia, and diabetes. The reasons for detecting a difference in performance among students who self-reported native versus non-native proficiency may include variations in self-reporting practices (e.g., some students may overestimate or underestimate their proficiency) or difference in the perceived complexity of the topic presented. For example, students at a more advanced proficiency level may have had more prior exposures to certain health topics than less fluent students who may not be as likely to have discussed those concepts in Spanish in the past. Of note, the first topic (“language and health overview”) displays twice the number of student survey responses since this topic is taught as the first session at the beginning of each academic year. Therefore, response data to knowledge questions about this topic are asked at the conclusion of the course after every academic year, whereas the focused health topic areas of the other sessions are different in year-one versus year-two of the curriculum (Table 1).

For future studies, the post-course data should be compared with an objective pre-course knowledge assessment and results should be compared to a control group of pre-health students who receive standard pre-medical courses without the supplemental Spanish health topics curriculum. In addition, the study’s self-reported proficiency and comfort level assessments may be limited in accuracy, and expanded pre- and post-assessment of students should be considered in future investigations to better understand both pre-existing knowledge base about specific topics, language proficiency, and effect of the educational intervention.

For most students, *Temas de Salud* represents an intervention that provides the first opportunity for formal learning of health and clinical information that has applicability and impact not only to their future career but also to their current lives, as evidenced by their usage of the information with their families, friends, and in other life contexts. Many students provided free-text feedback as part of their survey responses, and several commented that they valued gaining knowledge regarding Spanish medical terminology that they anticipate to be a necessity for them in their medical training. One student summarized that the course “introduced me to Spanish words I did not know but will need to know for my future career.”

Others commented on the importance of the cultural connection to their heritage language, such as the student who commented: “I had never before taken a course or listened to an academic lecture in my native language, so this exposure was definitely beneficial.” Another student mentioned “having discussions in Spanish, I really enjoyed that. I would probably never had the experience anywhere except here.” Many students made connections between content presented in the course and the ability to care for their family members and community. This is consistent with prior literature that underrepresented minority physicians are more likely to care for underserved populations, although the effect of physician's multilingual skills on practice location decisions has not been studied [19]. One *Temas de Salud* student wrote, “It definitely helped me to be able to communicate this to my own family and be more open-minded. I am actually looking forward to educating myself [further] on these topics in Spanish” indicating motivation to continue gaining language and cultural skills. Yet another student stated, “[In] college we don't really talk about health problems in [the] Latino community, [but throughTemas de Salud] every month, I am reminded why I am here, why I am studying, and why medicine was my passion, [to] help my underrepresented community.”

Anecdotal reports from *Temas de Salud* students suggest that after their experience in the course, participants are choosing to enrich their undergraduate careers such as adding double-majors, minors, or other coursework with the goal of improving their linguistic competence in Spanish. To our knowledge, Medicina Scholars is the first pre-professional pipeline program with a formal Spanish curriculum that is tracking student performance and comfort level in an effort to provide the evidence base to support the development of health-related language and cultural skill set in pre-medical under-represented students. Additional studies tracking students over time may help establish the long-term benefits of a Spanish Health Topics course for pre-medical undergraduates on target criteria such as matriculation in medical school, academic performance metrics, the likelihood of future practice in underserved communities, and student career satisfaction.

## Conclusions

The integration of health concepts through a Spanish-immersion program for Latino pre-health students can improve student comfort level, enhance knowledge acquisition, and promote culturally-relevant health concept applications to students’ social and academic environments. *Temas de Salud* is a pilot program that may serve as a model for pre-health training to increase the Spanish health language and culturally-relevant exposure and preparedness of Latino premedical undergraduates. Our course builds upon Latino students' heritage language skills and cultural experiences and enhances their skills as applied to medical contexts. Such strategies that build upon Latino or other underrepresented minorities’ pre-existing knowledge base may serve to validate their diverse lived experience as a valuable clinical asset that can be supported by formal educational efforts. *Temas de Salud* aims to empower Latino students to value and enhance their bilingual, bicultural health skillset early in their career path. As such, our program represents an opportunity for replication in other undergraduate institutions that may be interested in developing pipeline programs to promote underrepresented, multilingual minorities in medicine.

## Figures and Tables

**Figure 2:**
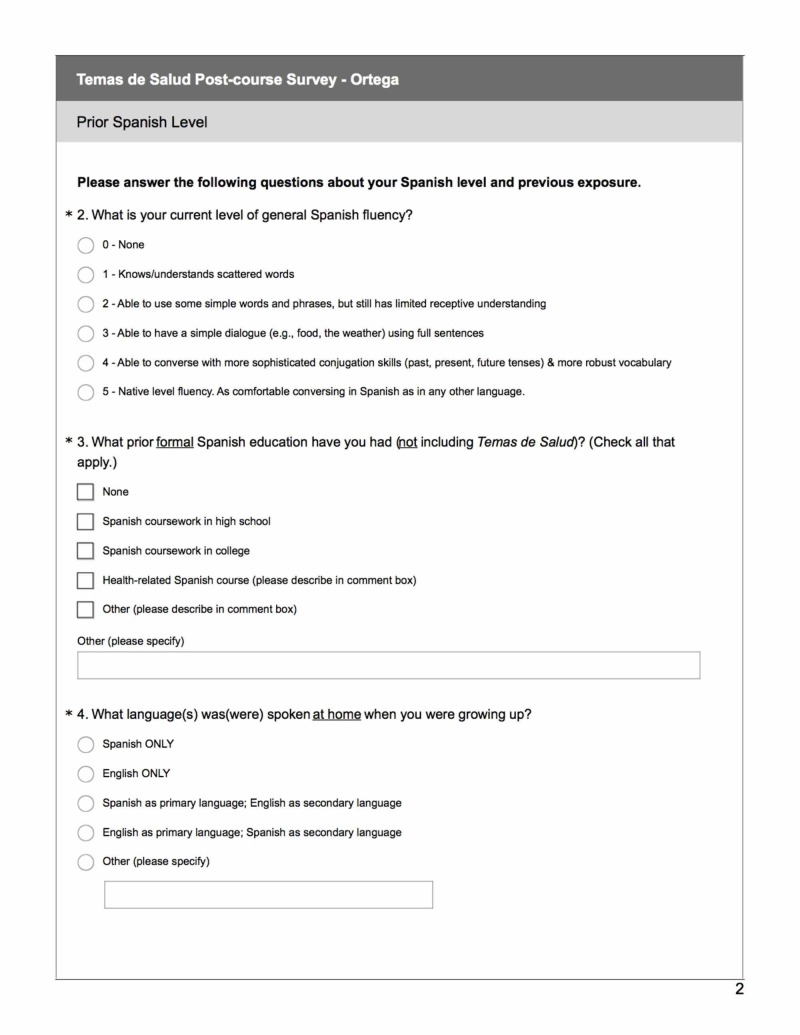
Selected Proficiency Questions from the Temas de Salud Assessment Instrument

**Figure 3:**
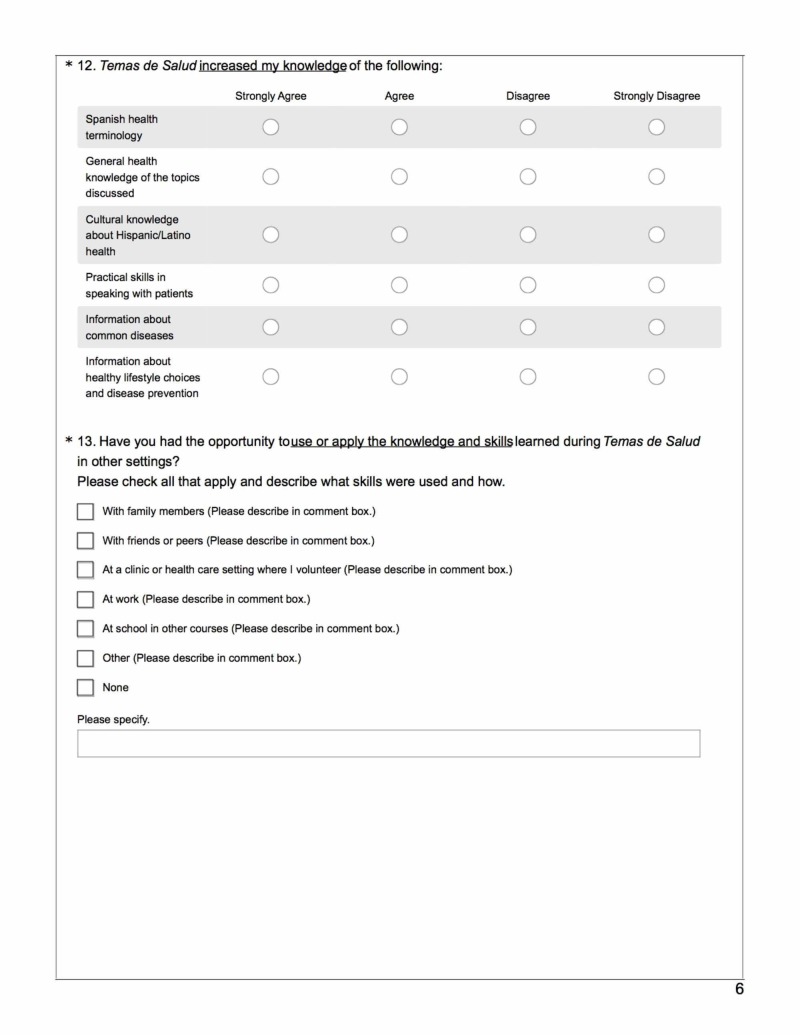
Selected Perceived Change in Knowledge Questions from the Temas de Salud Assessment Instrument
